# Racial and incident discrepancies in news media reporting of sudden unexpected infant death (SUID)

**DOI:** 10.1186/s40621-022-00398-2

**Published:** 2022-12-21

**Authors:** Sarah Gard Lazarus, Terri Miller, Philip J. Hudson, Terri McFadden, Gretchen Baas, Sadiqa Kendi

**Affiliations:** 1 Pediatric Emergency Medicine Associates, Atlanta, GA USA; 2 Children’s Healthcare of Atlanta, Atlanta, GA USA; 3 Georgia Department of Public Health, Atlanta, GA USA; 4 Emory University Department of Pediatrics, Atlanta, GA USA; 5 Rollins School of Public Health, Atlanta, GA USA; 6 Children’s National Hospital, Washington, DC USA

**Keywords:** Infant, Injury prevention, Pediatrics, Race, SIDS, Suffocation, SUID

## Abstract

**Background:**

Regardless of injury prevention and outreach efforts, there continue to be low rates of adherence with the American Academy of Pediatrics (AAP) safe sleep recommendations. Media is an important tool for parental education and may influence risk perception and caregiver choices. Due to media reports potentially serving as an opportunity for shaping social norms, caregiver education and injury prevention, an evaluation was undertaken to evaluate Georgia local news reporting of sudden unexpected infant death (SUID) as compared to drownings, homicides, and firearm injuries. Our objective was to evaluate incident and racial discrepancies in Georgia news media reporting of SUID as compared to other pediatric injury deaths.

**Results:**

Despite its high incidence, SUID was far less commonly mentioned in the news media, with only 1.9% (10/525) mentioned as compared to 8.1% of drownings (17/211), 11.4% (74/649) of MVC’s, 14.7% (59/402) of homicides between ages 1–18, 20% (11/55) of fire-related deaths and 25% (15/59) of homicides under age one (infant homicides). Across SUID and homicide, deaths of White infants were reported in the news media at 2.5 times the rate of Black infants.

**Conclusion:**

Despite SUID being a leading cause of infant death, it is infrequently mentioned in the news media. When mentioned, the news media are more likely to highlight the deaths of White infants as compared to Black infants, though the incidence rate of SUID is higher in Black infants as compared to White.

## Background

Unintentional injuries are the leading cause of childhood death. SUID, a leading cause of death under age 1, includes sudden infant death syndrome (SIDS), accidental suffocation and strangulation in bed (ASSB) and death from unknown causes (Centers for Disease Control and Prevention 2018; Moon 2016). There are significant disparities in mortality, with SUID rates impacting approximately twice as many Black infants as compared to White infants (Colson et al. 2017; Parks et al. 2017; Center for Disease Control and Prevention 2017). Regardless of previous injury prevention and outreach efforts, there continue to be low rates of adherence with the American Academy of Pediatrics (AAP) safe sleep recommendations (Colson et al. 2017; Parks et al. 2017; Center for Disease Control and Prevention 2017). Behavior change can be challenging, and people must feel uncomfortable with their current behavior in order to enact change, as theorized by the health belief model (Champion et al. 2008). Media is an important tool for parental education and may influence risk perception and caregiver choices. It may reinforce social norms or elevate one’s risk perception (Woloshin et al. 2017; Roehler et al. 2018; Yu et al. 2016). Media representations of safe sleep do not always align with AAP recommendations. More than one-third of images depicting infants sleeping in magazines geared to women of child-bearing age show inappropriate sleep position (Joyner et al. 2009; Epstein et al. 2011).

Due to media reports potentially serving as an opportunity for shaping social norms, caregiver education and injury prevention, an evaluation was undertaken to determine Georgia local news reporting of SUID as compared to drownings, homicides, and firearm injuries (Woloshin et al. 2017). In addition, these deaths were further analyzed to determine if race was correlated with news media reporting.

Our objectives were to:

1. Evaluate the proportion of pediatric injury deaths from various causes in news media reports and compare to the publicly available public health reporting database, the Georgia Department of Public Health Online Analytical Statistical Information System (GDPH OASIS) (Online Analytical Statistical Information System (OASIS) 2020).
2. Evaluate whether racial discrepancies exist in Georgia news media reports of pediatric injury deaths.

## Discussion

Despite its high incidence, SUID was far less commonly mentioned in the news media, with only 1.9% mentioned, as compared to 8.1% of drownings, 11.4% of MVC’s, 14.7% of homicides between the ages of 1–18, 20% of fire-related deaths and 25% of homicides under age one. When compared to SUID, these other deaths were mentioned in the news media between 4 and 13 times more. MVCs caused the largest number of injury deaths in the 0–18 year-old range from 2014 to 2018 in Georgia. However, SUID was almost as common and affected a much smaller age range, creating a proportionally larger population impact.

### Racial discrepancies

This study is the first to examine racial discrepancies in the news media reporting of SUID. In addition, it corroborates that there is a mismatch between news media reporting of SUID and incidence. In Chicago between 2011 and 2015, news media reported 42 (59.2%) of the MVC deaths, 17 (37.8%) of the fire-related deaths, and 0 (0%) of the SUID deaths. Despite there being more than twice the SUID deaths than the number of MVC and fire-related deaths combined, there was a lack of news media coverage, which may lead to lower risk perception (Roehler et al. 2018). News media reporting also infrequently provides prevention messaging, which may be a missed opportunity for education and outreach (Faulkenberry and Schaechter 2015; Leavy et al. 2019). It is unclear why SUID is so infrequently mentioned in the news media.

Social media and mobile health intervention are being utilized increasingly to educate parents about safe sleep and other injury prevention topics. In a recent study, mothers who received safe sleep videos via text message or email were more likely to both place their infant supine to sleep and to room share. Although this study did not look at racial disparities in depth, they did state that “baseline levels of nonadherence [to safe sleep practices] were lower in African Americans…the SS [safe sleep] mHealth intervention improved these rates to levels comparable to those of other groups” (Moon et al. 2019). As parents of all backgrounds become more dependent on social media platforms and mobile health information to learn about health topics, it is important that we provide accurate information and prevention recommendations, specifically regarding safe sleep (Huo et al. 2019).

Both SUID and infant homicide disproportionately affected Black infants, with 58% (297/512) of SUID deaths and 71% (41/58) of infant homicide affecting Black infants (Table 3). We specifically focused on infant homicide due to finding during our research that many deaths that were originally thought to be SUID after further investigation were actually classified as infant homicide. By separating infant homicide from homicide in ages 1–18, we were able to directly compare infant homicides to SUID and analyze race data in the under 1-year age group.

White infant deaths were reported in the news media at 2.5 times the rate of Black infant deaths, controlling for whether they died of SUID or homicide. The disproportionately lower news media reporting of these deaths may give parents and communities the false impression that these deaths do not occur or do not have as much of an impact. If communities are unaware of an issue, they are less likely to be mobilized to address the issue and it also reduces the ability to add prevention messaging.

Studies suggest that social inequalities, not individual behaviors, are the main reason for health disparities, including discrepancies in social, environmental, physical, and economic conditions (Link and Phelan 1995; Kindig 2007; Berkman and Kawachi 2000). Previous studies have shown that the coverage of racial and ethnic health disparities has decreased since 1998. It is believed that the declining coverage of racial and ethnic health disparities may explain why the public is not aware of these health disparities (Kim et al. 2010). At least one previous network television producer has stated that bias is often present in local television newsrooms, and it is difficult to study the degree to which minorities are omitted from local news coverage (Lipschultz and Race 2003). Lack of communication and transparency may contribute to health disparities (Viswanath et al. 2006; Wallington et al. 2010; Nagler et al. 2016). By omitting those who are affected most by SUID, we are missing an important opportunity for education and prevention.

### Limitations

During the evaluation of our news media reports, we had to rely on visual representation of race as compared to self-description, making us unable to determine ethnicity. Death records were not individually queried to determine the correlation of news media reports to the death record. News media reports were not linked to death records and all calculations were made using the separate news media report counts collected here and deaths as reported by OASIS. For SUID, OASIS counts only use the ICD-10 code R95 (SIDS) but exclude the related codes R99 (undetermined) and W75 (accidental suffocation and strangulation in bed) used to identify SUID cases. Because of this, the rates for news media reporting of SIDS may be overestimated here and the true disparities between the reporting of sleep related deaths and other injury related causes of death may be even greater. For homicides, many news media reports were not reported until someone was charged.

Race values were not available consistently in four out of our six categories (MVC, drowning, fire-related, and homicide between ages 1–18), as they were only reliably found for homicide under age one and SUID, so could only reliably be calculated for these two causes of death. Based on the significant number of small news media networks not affiliated with major cities and the countless ways that news is disseminated, our study was limited to local news networks and did not include print or social media. Despite limitations, this study provides an initial look at disparities in media reporting of injury deaths, particularly SUID. Future work will further evaluate racial disparities in news media reporting by linking individual news media reports to death records and evaluating findings in additional geographic areas.

## Conclusions

Our study highlights the lack of media reporting of SUID when compared to other pediatric causes of injury death. In addition, our study shows racial disparities in news media reporting of SUID, which to date has not been reported in the literature. Even though Black infants are more likely to die from SUID, deaths of white children under the age of one were reported in the news media at 2.5 times the rate of Black children under the age of one. Further study, with more robust data collection and linkage methodology, is needed to better understand and address this pattern.

## Methods

### Study design and data collection

After IRB exemption through Georgia Department of Public Health (DPH), the Google^©^ search database was utilized to query local news media affiliates of NBC, ABC, CBS and Fox television stations in the following Georgia cities; Atlanta, Augusta, Columbus, Savannah, and Albany. Each of the local news websites were then searched utilizing 31 search terms for each of the following injury areas: SUID, motor vehicle collisions (MVCs), drownings, fire-related deaths, homicide of ages 1–18 years and homicide under age 1 year (referred to as infant homicides). The timeframe for the various injury deaths covered a 5-year span with corresponding news media reports ranging from January first of 2014 through December thirty-first of 2018. These news media reports were compared to the Online Analytical Statistical Information System (OASIS), a verified public health reporting database through Georgia DPH. The OASIS data are recorded directly from Georgia death certificate records (Online Analytical Statistical Information System (OASIS) 2020). Table 1 contains the Google^©^ search terms used to identify news media reported cases as well as applicable ICD-9 and ICD-10 codes for each cause used in OASIS to identify total death counts. For all reports, a news media report was excluded if decedent was above 18 years, the death was outside of stated time range, or age was not listed, with the exception of infant deaths. Non-Georgia residents were excluded since OASIS data is specific to Georgia residents.

**Table 1:** Search terms and ICD-10 (ICD-9) codes used

Cause of death and age group	New media search terms	ICD-10 (ICD-9) codes^a^
SUID (< 1)	SIDS, SUID, suffocate (infant, GA), strangle, bedsharing, entrapment, infant death, suffocation	R95(798.0)
Homicide (< 1)	Infant death, homicide, cruelty, internal injury, blunt force trauma	X85-Y09, Y87.1 (E960-E969)
Homicide (1–18)	Homicide, cruelty, gunshot, teen dies, murder	X85-Y09, Y87.1 (E960-E969)
MVC (0–18)	Fatal crash, traffic death, teen driver, fatal accident, MVC, pedestrian, bicycle, cyclist, hit and run, car seat	V02-V04, V09.0, V09.2, V12-V14, V19.0-V19.2, V19.4-V19.6, V20-V79, V80.3-V80.5, V81.0-V81.1, V82.0-V82.1, V83-V86, V87.0-V87.8, V88.0-V88.8, V89.0, V89.2 (E810-E825)
Fire (0–18)	Deadly fire, arson, inhalation	X00-X09 (E890-E899)
Drowning (0–18)	Drown, drowning, lake, pool, drowns	W65-W74 (E910)

^a^Data Source: Georgia Department of Public Health Online Analytical Statistical Information System (GDPH OASIS)

When evaluating SUID, search terms had “infant, GA” added to help narrow the search to Georgia specific reports. If exact age was not mentioned, but the news media report mentioned “infant,” the report was included since this term is specific to a child under 1 year of age. If the news media reporting of the SUID was unclear, three medical professionals (two public health analysts and a physician TM, PJH, SGL) consulted together to determine if the report would be included based on known inclusion and exclusion criteria. If SUID was later deemed homicide after a trial, the news media report was excluded from SUID categorization and included in infant homicide. If the news media report was a duplicate, it was removed during the data-cleaning process.

### Statistical analysis

After collecting the counts of news media reported deaths and OASIS reports, percentages were calculated by cause and age group. Relative risk statistics were used to calculate relative rates of news media reporting, with 95% confidence intervals, to compare the difference in reporting of deaths by cause and age group. Because individual news reports were not linked to individual death records, when necessary for calculation, deaths not reported in the news media were derived as the difference between counts of OASIS reports and news media reported deaths. During data collection, investigators attempted to identify decedent race through the initial news media reports as well as any associated obituaries. Hispanic ethnicity could not reliably be identified as it was not mentioned in the news media reports. Race could not reliably be identified in most of the news media report categories except in two groups of interest: SUID and infant homicides. Due to this, further analysis looked at the relationship between child race and media reporting using the Breslow-Day test of homogeneity and the Cochran–Mantel–Haenszel test of general association with control for whether the cause of death was SUID or homicide. A common relative risk estimate was generated to describe the relationship between race and media reporting, adjusting for the cause of death. Data analysis was conducted in SAS 9.4.

## Results

Counts and rates of news media reporting by selected cause and age groups, as well as relative rates of news media reporting compared to SUID are presented in Table 2. Deaths in each cause and age group of interest (including infant homicide, homicide ages 1–18, MVC ages 0–18, fire ages 0–18, and drowning ages 0–18) were separately reported in the news media at significantly higher rates compared to SUID. Infant homicides were reported at the highest rate relative to SUID, being reported in the news media at 13.3 [95% CI 6.3–28.3] times the rate of SUID.

**Table 2:** Rates of media reported deaths by cause and age, with relative rates of reporting compared to SUID deaths, Georgia, USA, 2014–2018

Cause of death and age group	Media reported deaths (*n*)	Total deaths (*n*)^a^	Media reported deaths per 100 total deaths (%)	Relative rate of reporting [Wald 95% CI]
SUID (< 1)	10	524	1.9	Ref.
Homicide (< 1)	15	59	25	13.3 [6.3–28.3]
Homicide (1–18)	59	402	14.7	7.7 [3.9–14.9]
MVC (0–18)	74	649	11.4	5.9 [3.1–11.5]
Fire (0–18)	11	55	20	10.5 [4.7–23.6]
Drowning (0–18)	17	211	8.1	4.2 [1.9–9.1]

^a^Data Source: Georgia Department of Public Health Online Analytical Statistical Information System (GDPH OASIS)

Only SUID and infant homicides had complete race data and therefore, were subject to further analysis. News media reported deaths, OASIS reported deaths, and percentage of total deaths reported for White children and Black children in the SUID and infant homicide categories are presented in Table 3**.** The test results of the statistical analysis are presented in Table 4. The results of the Breslow-Day test of homogeneity indicated the relationship between child race and news media reporting was consistent across infant homicide and SUID. According to the results of the Cochran–Mantel–Haenszel test of general association, child race and news media reporting were not independent. Adjusted for cause of death, White infant deaths were reported in the media at 2.50 (95% CI [1.20, 5.20]) times the rate of Black infant deaths.

**Table 3:** Rates of media reported deaths by cause and race among infants, Georgia, USA, 2014–2018

Cause of death and age group	Child race (any ethnicity)	Media reported deaths (*n*)	Total deaths^a^ (*n*)	Media reported deaths per 100 total deaths (%)
SUID (< 1)	White	7	215	3.3
	Black or African American	3	297	1.0
Homicide (< 1)	White	7	17	41.2
	Black or African American	8	41	19.5

^a^Data Source: Georgia Department of Public Health Online Analytical Statistical Information System (GDPH OASIS)

**Table 4:** Statistical test results

Test	${\chi }^{2}$	*df*	*p*
Breslow-Day test of homogeneity	.02	1	.887
Cochran–Mantel–Haenszel test of general association	6.16	1	.013*

*Statistically significant at the *p* < .05 level

## Data Availability

Data are publicly available via the OASIS database through the Georgia Department of Public Health at https://oasis.state.ga.us/. Further data are made available in figures and tables discussed in the manuscript.

## Abbreviations

AAP: American Academy of Pediatrics
ASSB: Accidental suffocation and strangulation in bed
DPH: Department of Public Health
MVC: Motor vehicle collision
OASIS: Online Analytical Statistical Information System
SIDS: Sudden infant death syndrome
SUID: Sudden unexpected infant death
